# Outcomes of idebenone therapy for Leber hereditary optic neuropathy in a cohort of patients from Wales

**DOI:** 10.1038/s41433-025-03993-x

**Published:** 2025-09-17

**Authors:** Francis W. B. Sanders, Marcela Votruba

**Affiliations:** 1 University Hospital of Wales, Cardiff, UK; 2 School of Optometry & Vision Sciences, Cardiff University, Cardiff, UK

**Keywords:** Outcomes research, Chemotherapy

## Abstract

**Background:**

Leber hereditary optic neuropathy (LHON) poses a significant burden to patients, with the majority not showing significant spontaneous improvement in their vision. The recent validation of idebenone as a therapy provides some avenue for benefit for patients with LHON in Wales, where it has been approved for use within the NHS.

**Methods:**

From March 2021, all patients being seen in a tertiary referral clinic and diagnosed with LHON by targeted genetic testing were offered treatment with idebenone and commenced on idebenone as part of standard care. For such patients, their clinical records were used to collect demographic and outcome data. Visual acuity was analysed for clinically relevant recovery (CRR), defined as improvement from ‘off-chart’ to ‘on-chart’ or ‘on-chart’ improvement of at least 10 letters.

**Results:**

A total of 12 (67% male) individuals were treated with idebenone 300 mg TDS for LHON for a mean period of 30.2 (±9.9) months. Mean visual acuity at initiation of therapy was 2.22 (±0.32) LogMAR, improving to a peak of 1.12 (±0.77) LogMAR at 27 months. This time point was coincident with the maximum CRR achieved, with 86% demonstrating CRR. At 24 months, CRR was significantly higher when compared to a natural history cohort.

**Conclusion:**

The present cohort demonstrates evidence of CRR in a high proportion of patients reaching 27 months of treatment. Further follow-up and a larger cohort of patients will provide further insight into the real-world efficacy of idebenone in LHON.

## Introduction

Leber hereditary optic neuropathy (LHON) is a rare disease but one of the most commonly described hereditary optic neuropathies, with a prevalence of 1 in 31,000 to 1 in 68,000 [1–4]. Although more recent analysis suggests a carrier rate of up to 1 in 1000 of the most common causative genetic variants, which highlights the incomplete penetrance demonstrated with those carrying the three most common causative variants, with only 2.5–17.5% of these carriers progressing to express the LHON phenotype [5–7]. The three most common variants in the mitochondrial genome—m.3460G>A (MTND1), m.11778G>A (MTND4), and m.14484T>C (MTND6)—are identified as the causative variant in over 95% of LHON cases [8, 9].

LHON is characterised by centrocecal vision loss that can occur at any age but typically has two peaks of incidence in early adulthood and a smaller increase in incidence around late middle age [8, 10]. Individuals tend to experience profound visual loss, which is limiting on their daily activities and often adversely impacts their ability to work [11]. There is, however, some evidence for spontaneous improvement of visual function over months to years following the initial decline in vision [9]. This is reported to be more common amongst certain variants, particularly the m.14484T>C variant, and less frequently observed in the m.11778G>A and m.3460G>A variants [4, 9, 12–14].

Up until recently, there have been very limited management options beyond maximising residual vision with magnifying aids and supportive measures to improve quality of life [10, 11]. More recently, several novel therapies have been trialled, from oral supplements and medications to genetic therapies [15–17]. Of particular interest is oral idebenone dosed at 300 mg three times daily, which was approved by the European Medicines Agency and has been established in several European nations as standard therapy following a number of trials, including the RHODOS study and the LEROS study [3, 13, 18–20].

Briefly the LEROS trial demonstrated benefits for those patients dosed with idebenone in terms of their clinically relevant recovery (CRR) deemed as improvement of 10 letters on-chart or from off-chart to a minimum of 5 letters on-chart, with 46.0% demonstrating CRR when compared to those on placebo in the extended access programme and 31.9–47.9% demonstrating CRR in the LEROS trial depending on when therapy was initiated [3, 13]. Based on this evidence, idebenone was recommended for use in the National Health Service in Wales for patients with LHON by the All Wales Medicines Strategy Group (AWMSG) in their final appraisal recommendation (Advice No: 0521—March 2021) [21].

The present study presents an overview of the outcomes of all patients treated with idebenone within the tertiary referral ophthalmic genetics clinic in University Hospital of Wales (UHW), Cardiff, following their initiation of treatment from 2021 up until January 2025.

## Methods

Patients attending the tertiary referral clinic for ophthalmic genetics within UHW in Cardiff with a diagnosis of LHON were identified. All patients were offered treatment as part of their routine clinical care in line with prescribing guidelines and authorisation in line with AWMSG advice. Idebenone was prescribed at 300 mg orally three times daily (TDS). For comparison to a natural history cohort (NH), data were extracted from previous published datasets of patients with LHON used in the LEROS nonrandomised controlled trial, which has previously been described [13]. This was extracted from two international, multicentre historical case record surveys CRS-1 (ClinicalTrials.gov: NCT01892943) and CRS-2 (ClinicalTrials.gov: NCT02796274).

Demographic data were collected from patients’ medical records, as well as details of diagnosis and the outcome of genetic testing and data on visual outcomes. All patients were confirmed to have an established genetic diagnosis of LHON associated with one of the three primary variants in mitochondrial DNA by NHS genetic testing in the form of targeted gene sequencing, next-generation based gene panel, or mitochondrial gene sequencing. All data capture was finalised as of 29th January 2025.

Visual acuity data were collected and converted to LogMAR units where not collected in this format. This was performed using the previously published calculator [22] based on the United Kingdom National Ophthalmology Database published values [23]. From this, CRR was defined as improvement from ‘off-chart’ to ‘on-chart’ or ‘on-chart’ improvement of at least 10 letters. This was adapted from previous studies of idebenone therapy in LHON [13, 18, 19], given the mixed methods of visual acuity assessment from ‘off-chart’ to ‘on-chart’ and subsequent conversion of some visual acuity outcomes recorded in metric Snellen format to LogMAR equivalent outcomes.

Statistical analysis was performed using SPSS (Version 29.0.2.0, 2023, IBM, Armonk, NY, USA). Continuous unpaired parametric data were compared using a two-sided Student’s *t*-test, whilst paired non-parametric data were compared using Friedman’s two-way analysis of variance by ranks with Dunn’s post hoc analysis and Bonferroni correction for multiple comparisons. Fisher’s exact test was performed for categorical data with expected counts of less than 10, with subsequent logistic regression controlling for known a priori characteristics. Significance was defined at the level of *p* < 0.05. Continuous data are presented as mean ± standard deviation and categorical data comparison is summarised as an odds ratio with a 95% confidence interval.

This study was approved locally (Cardiff & Vale University Health Board Research & Development) and registered on the local Audit Management and Tracking system. It was conducted in accordance with the tenets of the Declaration of Helsinki.

## Results

A total of 29 individuals with mitochondrial variants associated with LHON were identified in the ophthalmic genetics service. Six tested positive for the m.14484T>C variant and the remaining 23 tested positive for the m.11778G>A variant.

All patients were offered treatment, excluding those who had experienced prior spontaneous visual recovery or exhibited LHON with ‘plus’ features, such as one female patient with Harding’s syndrome. No restriction was placed on time from vision loss, although a number of those with the longest potential durations of disease were either not being routinely followed up, or had been lost to follow-up, or had been discharged some years before. Thus, 12 were initiated on idebenone therapy under the NHS ophthalmic genetics clinic in UHW in accordance with the AWMSG guidance. All were offered six-monthly appointments to monitor vision and any side effects. One patient was excluded from the current analysis as they were restarted on therapy for only 6 months, having previously been treated within the LEROS trial [13]. There were 8 males and 4 females, with an average age when initiating therapy of 47.2 (±19.3) years, ranging from 22 to 72 years of age. Prior to starting idebenone, the mean duration of symptoms attributed to LHON was 31.5 (±59.5) months with a total range from 2 to 216 months. Four patients tested positive for the m.14484T>C pathogenic variant, and the remaining eight carried the m.11778G>A variant. Two treated patients had a known family history of LHON, one with the m.14484T>C variant and one carried the m.11778G>A variant. The remaining 10 patients had no clear family history and were suspected de novo variants, albeit not all family members have undergone genetic testing to investigate their carrier status.

The NH cohort comprised 346 eyes reaching 12 months of follow-up and 168 eyes achieving 24 months of follow-up. NH cohort patients had an average age of 31.7 (±15.0) years for those achieving 12 months of follow-up and 29.7 (±14.8) years for those achieving 24 months of follow-up. These were both significantly different from the treated patients in the current cohort (*p* < 0.001). Male individuals accounted for 81% of eyes reaching 12 months in the NH cohort and 83% of those reaching 24 months of follow-up, neither of which was significantly different from the treated cohort presented herein (12 months v treated, *p* = 0.134; 24 months v treated, *p* = 0.287). In terms of the proportion of patients in the subacute/dynamic phase of the disease versus the chronic phase of the disease (as defined previously as <1 year from disease onset for subacute/dynamic or >1 year for chronic) [13], this was not significantly different from the current treated Welsh cohort in comparison to the NH cohort at either the 12-month time point after starting treatment/follow-up (idebenone 56% v NH 56%, *p* = 1.00) or the 24-month time point after starting treatment/follow-up (idebenone 57% v NH 45%, *p* = 0.412).

The cohort treated with idebenone had a mixed history of alcohol and tobacco usage. Six reported smoking more than 10 cigarettes a day, although two of these subsequently ceased smoking after their diagnosis of LHON. Four of the cohort reported drinking 10 or more units of alcohol per week, with two reporting heavy use of 40 units or more. Of the entire cohort, no mention was made of significant tobacco usage or alcohol consumption in the case of three individuals, whereas two individuals reported zero tobacco smoking or alcohol consumption.

At the point of data collection, the patients had received an average of 30.2 (±9.9) months of therapy, ranging from 14 to 42 months in total. No side effects were reported. Two patients were excluded at this point of analysis due to being lost to follow-up, leaving 10 patients continuing therapy. Additionally, one patient died whilst taking idebenone of coexistent medical comorbidities after 13 months of therapy. Two patients had ceased therapy after 30 and 35 months, respectively, by mutual consent on clinical review. One ceased due to no response over the 30 months of therapy. The second individual demonstrated CRR between 21 and 30 months; however, by 35 months, their vision deteriorated to baseline in both eyes and they elected to cease idebenone therapy.

**Fig. 1:**
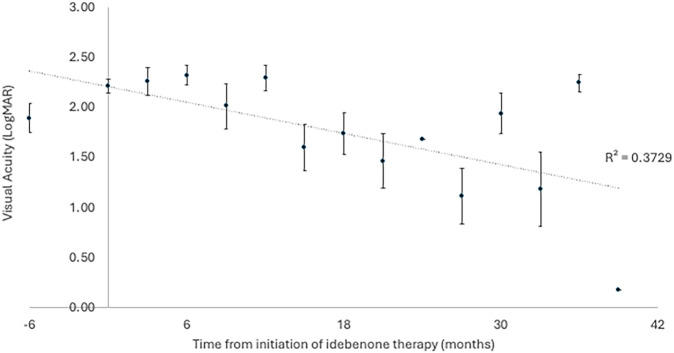
The mean visual acuity (LogMAR) of 12 patients with LHON treated with idebenone over time (months). Displayed as mean ± standard error of the mean.

**Fig. 2:**
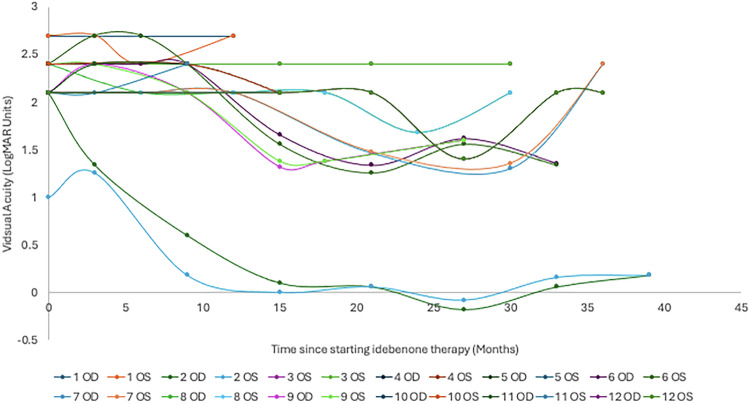
The measured visual acuity (LogMAR) of each eye in all 12 patients treated with idebenone over time (months). OD—right eye; OS—left eye.

The primary outcome of the study was visual acuity, with patients having a mean visual acuity equivalent to 1.89 (±0.71, 24 eyes) LogMAR units at diagnosis of LHON. At the time of therapy initiation (0 months), the patient’s vision had deteriorated to 2.22 (±0.32, 24 eyes) LogMAR units. The most recent visual acuity for the 7 patients still receiving idebenone therapy is 1.69 (±0.72, 14 eyes) LogMAR units. The peak mean visual acuity within the first 36 months of therapy was at 27 months, which was 1.12 (± 0.77, 8 eyes) LogMAR units for the limited patients with a review at this time point. The time course of visual acuity is illustrated in Figs. 1 and 2.

In line with the previously published LEROS study [13], comparison was made between visual acuity outcomes at 0 months, 12 months and 24 months of therapy. There was no difference between 0 months (2.22 ± 0.32) and 12 months (2.11 ± 0.62; *p* > 0.05). Whereas there was significant improvement in vision after 24 months of therapy (1.48 ± 0.72) from both 0 months of therapy (Bonferroni adjusted *p* = 0.042) and 12 months of therapy (Bonferroni adjusted *p* = 0.004).

**Fig. 3:**
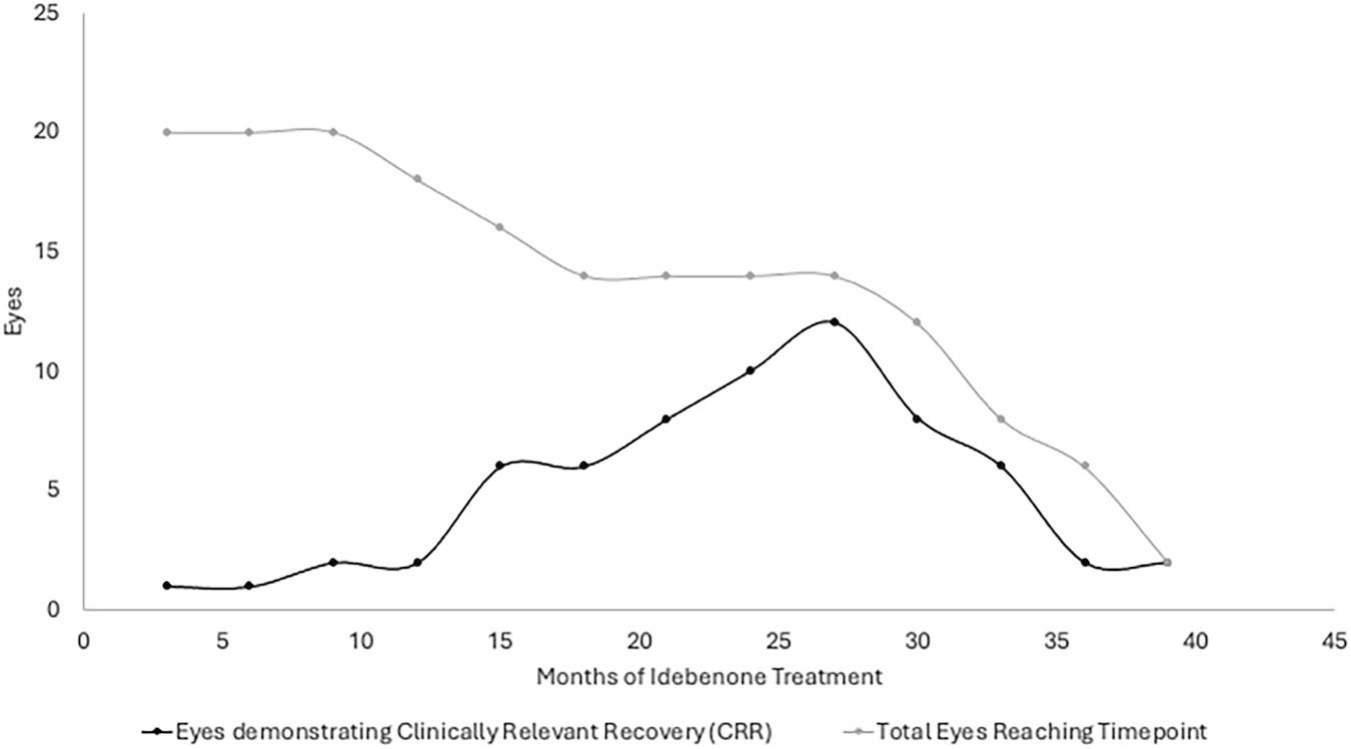
Number of eyes within the cohort of patients treated with idebenone demonstrating clinically relevant recovery (CRR) at each time point (months). The total number of eyes within the cohort that reach each time point after initiation is represented to demonstrate the relative proportion of eyes reaching a time point that demonstrate CRR. CRR is defined as improvement in vision from ‘off-chart’ to at least 1 letter ‘on-chart’, or improvement of at least 10 letters ‘on-chart’.

In terms of CRR, 10% of a total of 10 patients (20 eyes) reaching 3 months of therapy demonstrated CRR. This increased to a peak of 86% of 7 patients (14 eyes) reaching 27 months of therapy, but declined thereafter with far fewer patients (3 patients, 6 eyes) reaching beyond 34 months of therapy. This is outlined in Fig. 3.

The 12- and 24-month CRR outcomes were compared with the NH cohort. At 12 months, there was no significant difference in CRR rates between the treated group and the NH group (idebenone 11% v NH 19%; *p* = 0.545). However, at 24 months, CRR was significantly higher in the Welsh cohort treated with idebenone compared to the NH cohort (idebenone 71% v NH 24%; *p* < 0.001). To investigate this further, logistic regression analysis was performed to assess the effect of sex, age, and treatment with idebenone on the likelihood of achieving CRR at 24 months following treatment initiation or follow-up. The model was statistically significant when compared to the null model (*χ*^2^ (3) = 29.243, *p* < 0.001), explaining 21.3% of variation in CRR (Nagelkerke *R*^2^) and predicting 76.9% of cases correctly. Both treatment with idebenone (OR 0.105, 95% CI 0.021 to 0.532; *p* = 0.008) and a younger age (OR 0.737, 95% CI 0.589 to 0.923; *p* = 0.006) were significant in contributing to this model; however, an individual’s sex was not (OR 1.117, 95% CI 0.454 to 2.748; *p* = 0.810).

The individual patient who demonstrated CRR at 3 months has continued to improve up until 27 months with a peak visual acuity of −0.18 LogMAR units in the right eye and −0.08 LogMAR units in the left eye. This has, however, deteriorated up to 39 months, where their vision was 0.18 LogMAR units in the right eye and 0.18 LogMAR units in the left eye. This is relative to a visual acuity of counting fingers (equivalent to 2.1 LogMAR units) in the right eye and 1.0 LogMAR units in the left eye at the point of initiating therapy.

Examining the visual field tests of this individual demonstrated some improvement in terms of visual field index (VFI) and mean deviation (MD) on Humphrey 24-2 visual field testing using the SITA standard protocol. There was only a full-field 120-point suprathreshold test available prior to treatment initiation, which demonstrated a centrocecal scotoma within the central 20 degrees of both eyes. At 9 months after therapy initiation, the individual achieved a VFI of 75% in the left eye and 59% in the right eye and a MD of −8.39 dB in the left eye and −13.00 dB in the right eye. After a further 13 months of therapy, at 22 months total, they achieved a VFI of 77% in the left eye and 66% in the right eye, as well as an MD of −7.50 dB in the left eye and −10.09 dB in the right eye.

Three cases were identified as developing vision loss with no previous visual symptoms in older male patients (63, 68 and 72 years of age, all from separate families) with the m.14484T>C pathogenic variant coincidentally subsequent to COVID-19 vaccines. All three of these individuals started on idebenone within 4–8 months of the onset of symptoms. All three also demonstrated CRR at some time point during their treatment between 15 and 33 months. However, at the most recent review, one had ceased therapy due to completing 36 months of treatment and not retaining CRR. Meanwhile, the others continue therapy, although one individual’s vision has returned to baseline and the other retains some vision at 1.36 and 1.34 LogMAR units in the right and left eyes, respectively.

## Discussion

The present cohort summarises the clinical outcomes of 12 patients treated with idebenone for LHON. In the somewhat heterogeneous environment of real-world treatment, there is a surprising degree of CRR within the cohort, with 86% (10 of 14 eyes) achieving this by 27 months. This is a somewhat long time period of treatment to gain maximum response, given that 5 individuals had not reached 27 months of therapy. It does, however, correlate with other observations of clinical response up to 2 years [13, 19]. In comparison, the time course observed in the previous LEROS trial was briefer [13].

Previous reports have highlighted the ongoing efficacy of idebenone therapy within patients started on idebenone therapy in the acute phase (less than 1 year from disease onset), in the early chronic phase (1–5 years from disease onset) or in the late chronic phase (>5 years from disease onset) [24, 25]. In the current cohort, there were 6 (50%) patients treated within the acute phase, of whom 4 (67%) demonstrated CRR at any time point. Five (42%) patients were treated within the early chronic phase, only one of whom demonstrated CRR at any time point; however, two of these patients had no further clinical details beyond baseline as they were lost to follow-up, and one individual had only been on therapy for 12 months at the time of data collection. Only one (8%) patient was treated within the late chronic phase, who did demonstrate CRR.

Of interest, one individual who demonstrated CRR attained this despite initiating idebenone therapy with a significant time period since disease manifestation of 18 years. This is outside the time limit defined by the LEROS trial of individuals being within 5 years of diagnosis [13] or the Extended Access Programme study, which had more stringent restrictions of patients being within 1 year of disease onset [3, 19]. This does not contravene current prescribing recommendations of the AWMSG, which do not set a time limit of disease duration before starting therapy. This individual achieved CRR by 15 months and continues to demonstrate sustained improvement in VA up until their latest review at 27 months of therapy. This supports the finding in the LEROS trial suggesting VA improvement can continue to start until 24 months [3, 13, 24].

The presented cohort notably had solely 12 individuals with LHON treated with idebenone, in spite of a pool of 29 individuals being identified within the population served by the clinic as carrying pathogenic variants causative of LHON. One individual was ineligible due to exhibiting ‘plus’ features consistent with Harding’s syndrome. This presentation of LHON currently does not have evidence supporting the use of idebenone. Additionally, this includes those who may not benefit from idebenone because they have already experienced some spontaneous improvement or partial recovery of visual function, as was observed with approximately 30–40% of individuals within LHON in previous trials of idebenone [3, 13, 18, 19]. Furthermore, of the 17 individuals identified within the clinic as carrying pathogenic variants who did not start idebenone, 2 did not demonstrate any expression of the disease phenotype, 3 declined treatment through the clinic in UHW, 2 moved outside of Wales, and the remaining 10 did not respond to invitations to attend the clinic to consider treatment. In the presented cohort of patients, it is unclear who may have exhibited spontaneous visual recovery if idebenone had been omitted, especially given the atypical presentation of three older male individuals who started treatment relatively early in their disease’s time course. This may somewhat bias the results presented herein, either due to more or fewer patients experiencing CRR than might have in the placebo groups of prior LHON trials due to underlying differences in the demographics and clinical presentations of those with LHON [3, 8, 9].

When compared to the NH cohort established in the previously published LEROS study, the current cohort demonstrated improved CRR at the 24-month time point but not at the 12-month time point. The logistic regression model demonstrated that treatment and younger age were significant factors influencing a patient’s outcome in terms of CRR. Interestingly, given that the treated cohort presented herein was significantly older than the NH cohort, it would suggest that the treatment effect may have a greater influence on visual recovery at the 24-month time point. However, it is important to acknowledge that it is impossible to differentiate between the true treatment effect of idebenone or whether these patients were experiencing higher rates of spontaneous visual recovery than the NH cohort.

A limitation of the current study is the measurement of VA using a mixture of both LogMAR and Snellen VA in the clinic setting, which has subsequently been converted to LogMAR for analysis. Historically, before idebenone was a therapeutic option, the patients had Snellen acuities as a routine, and increasingly, all were having LogMAR acuity as they returned for follow-up. This has resulted in a subtly different definition of CRR in the presented study, which might allow for a higher reporting rate of CRR compared to those defined in LEROS, EAP and prior trials [13, 19]. That being said, in the context of the presented study reporting real-world outcomes and the difficulties of how VAs are measured in the clinic setting, where it may be the case that less encouragement of patients to attain a ‘better’ score compared to when those measuring VA have knowledge of an individual being a ‘trial’ patient and hence may prompt the participant to achieve a higher VA regardless of the test being used.

In conclusion, the presented real-world cohort of patients treated with oral idebenone over a period of 14–42 months demonstrates rates of CRR comparable to those reported in the previously published trials of idebenone therapy in people with LHON. This being a report of real-world outcomes in idebenone therapy, it is notable that such reports often demonstrate worse outcomes in terms of drug effectiveness when compared to observed drug efficacy when the therapy is applied in a controlled trial setting: the established concept of the ‘efficacy-effectiveness gap’ [26–28]. This is due to factors that influence the real-world clinic setting more so than a controlled trial: patients being lost to follow-up, dying, reduced motivation of clinic staff as compared to research staff, and less rigorous data collection with more flexible timepoints due to constraints on clinic capacity and staff availability. Therefore, further data collection from real-world observational studies is required to elucidate the true real-world effectiveness, the optimal timeframe of therapy initiation, as well as the duration of therapy that continues to provide clinically relevant benefit to patients with LHON [29].

## Summary

### What was known before

- Leber hereditary optic neuropathy is a debilitating condition resulting in severe visual disability, with emerging new therapies.
- Prior clinical trials have demonstrated the efficacy of idebenone in treating patients with Leber hereditary optic neuropathy and providing some meaningful improvement in visual function.

### What this study adds

- Real-world outcomes from treatment with oral idebenone in a cohort of patients with a diverse background show clinically relevant recovery of visual acuity in a majority of patients reaching 27 months of therapy.
- The presented study provides evidence for an extended course of therapy with idebenone, potentially to continue beyond 3 years, given that the recovery in visual acuity did not present in some individuals until the 27-month time point.

## Data Availability

All data generated or analysed during this study are included in this published article [and its supplementary information files].

## References

[1] Man PYW, Griffiths PG, Brown DT, Howell N, Turnbull DM, Chinnery PF. The epidemiology of Leber hereditary optic neuropathy in the North East of England. Am J Hum Genet. 2003;72:333–9. 12518276 10.1086/346066 PMC379226

[2] Puomila A, Hämäläinen P, Kivioja S, Savontaus ML, Koivumäki S, Huoponen K, et al. Epidemiology and penetrance of Leber hereditary optic neuropathy in Finland. Eur J Hum Genet. 2007;15:1079–89. 17406640 10.1038/sj.ejhg.5201828

[3] Klopstock T, Zeng LH, Priglinger C. Leber’s hereditary optic neuropathy—current status of idebenone and gene replacement therapies. Med Genet. 2025;37:57–63. 39963374 10.1515/medgen-2024-2066 PMC11831234

[4] Mascialino B, Leinonen M, Meier T. Meta-analysis of the prevalence of Leber hereditary optic neuropathy mtDNA mutations in Europe. Eur J Ophthalmol. 2012;22:461–5. 21928272 10.5301/ejo.5000055

[5] Mackey DA, Ong JS, MacGregor S, Whiteman DC, Craig JE, Lopez Sanchez MIG, et al. Is the disease risk and penetrance in Leber hereditary optic neuropathy actually low?. Am J Hum Genet. 2023;110:170–6. 36565701 10.1016/j.ajhg.2022.11.014 PMC9892764

[6] Mackey DA, Staffieri SE, Lopez Sanchez MIG, Kearns LS. Family and genetic counseling in Leber hereditary optic neuropathy. Ophthalmic Genet. 2025;46:101–9. 10.1080/13816810.2025.2451175 39833125

[7] Lopez Sanchez MIG, Kearns LS, Staffieri SE, Clarke L, McGuinness MB, Meteoukki W, et al. Establishing risk of vision loss in Leber hereditary optic neuropathy. Am J Hum Genet. 2021;108:2159–70. 34670133 10.1016/j.ajhg.2021.09.015 PMC8595929

[8] Poincenot L, Pearson AL, Karanjia R. Demographics of a large international population of patients affected by Leber’s hereditary optic neuropathy. Ophthalmology. 2020;127:679–88. 31932089 10.1016/j.ophtha.2019.11.014

[9] Yu-Wai-Man P, Griffiths PG, Chinnery PF. Mitochondrial optic neuropathies—disease mechanisms and therapeutic strategies. Prog Retin Eye Res. 2011;30:81–114. 21112411 10.1016/j.preteyeres.2010.11.002 PMC3081075

[10] Stramkauskaitė A, Povilaitytė I, Glebauskienė B, Liutkevičienė R. Clinical overview of Leber hereditary optic neuropathy. Acta Med Litu. 2022;29:9–18. 36061944 10.15388/Amed.2022.29.1.19 PMC9428633

[11] Kirkman MA, Korsten A, Leonhardt M, Dimitriadis K, De Coo IF, Klopstock T, et al. Quality of life in patients with leber hereditary optic neuropathy. Investig Ophthalmol Vis Sci. 2009;50:3112–5. 19255150 10.1167/iovs.08-3166

[12] Harding AE, Riordan-Eva P, Govan GG. Mitochondrial DNA diseases: genotype and phenotype in Leber’s hereditary optic neuropathy. Muscle Nerve Suppl. 1995;3:S82–4. 7603533 10.1002/mus.880181417

[13] Yu-Wai-Man P, Carelli V, Newman NJ, Silva MJ, Linden A, Van Stavern G, et al. Therapeutic benefit of idebenone in patients with Leber hereditary optic neuropathy: the LEROS nonrandomized controlled trial. Cell Rep Med. 2024;5:101437. 38428428 10.1016/j.xcrm.2024.101437 PMC10982982

[14] Newman NJ, Carelli V, Taiel M, Yu-Wai-Man P. Visual outcomes in Leber hereditary optic neuropathy patients with the m.11778G>A (MTND4) mitochondrial DNA mutation. J Neuroophthalmol. 2020;40:47. 10.1097/WNO.0000000000001045 32969847

[15] Chen BS, Yu-Wai-Man P, Newman NJ. Developments in the treatment of Leber hereditary optic neuropathy. Curr Neurol Neurosci Rep. 2022;22:881–92. 36414808 10.1007/s11910-022-01246-y PMC9750907

[16] Hage R, Vignal-Clermont C. Leber hereditary optic neuropathy: review of treatment and management. Front Neurol. 2021;12:651639. 34122299 10.3389/fneur.2021.651639 PMC8187781

[17] Chen BS, Newman NJ. Clinical trials in Leber hereditary optic neuropathy: outcomes and opportunities. Curr Opin Neurol. 2025;38:79. 39704163 10.1097/WCO.0000000000001343

[18] Klopstock T, Yu-Wai-Man P, Dimitriadis K, Rouleau J, Heck S, Bailie M, et al. A randomized placebo-controlled trial of idebenone in Leber’s hereditary optic neuropathy. Brain. 2011;134:2677–86. 21788663 10.1093/brain/awr170 PMC3170530

[19] Catarino CB, von, Livonius B, Priglinger C, Banik R, Matloob S, et al. Real-world clinical experience with idebenone in the treatment of Leber hereditary optic neuropathy. J Neuroophthalmol. 2020;40:558–65. 32991388 10.1097/WNO.0000000000001023 PMC7657145

[20] Raxone. European Medicines Agency (EMA) [Internet]. 2015 [cited 2025 Apr 2]. Available from: https://www.ema.europa.eu/en/medicines/human/EPAR/raxone.

[21] All Wales Therapeutics and Toxicology Centre [Internet]. [cited 2025 Apr 2]. idebenone (Raxone®). Available from: https://awttc.nhs.wales/accessing-medicines/medicine-recommendations/idebenone-raxone/.

[22] Moussa G, Bassilious K, Mathews N. A novel excel sheet conversion tool from Snellen fraction to LogMAR including ‘counting fingers’, ‘hand movement’, ‘light perception’ and ‘no light perception’ and focused review of literature of low visual acuity reference values. Acta Ophthalmol. 2021;99:e963–5. 33326177 10.1111/aos.14659

[23] Day AC, Donachie PHJ, Sparrow JM, Johnston RLRoyal College of Ophthalmologists’ National Ophthalmology Database The Royal College of Ophthalmologists’ National Ophthalmology Database study of cataract surgery: report 1, visual outcomes and complications. Eye. 2015;29:552–60. 25679413 10.1038/eye.2015.3 PMC4816350

[24] Pemp B, Kircher K, Reitner A. Visual function in chronic Leber’s hereditary optic neuropathy during idebenone treatment initiated 5 to 50 years after onset. Graefes Arch Clin Exp Ophthalmol. 2019;257:2751–7. 31482278 10.1007/s00417-019-04444-6

[25] Pemp B, Mitsch C, Kircher K, Reitner A. Changes in visual function and correlations with inner retinal structure in acute and chronic Leber’s hereditary optic neuropathy patients after treatment with idebenone. J Clin Med. 2021;10:151. 33406801 10.3390/jcm10010151 PMC7795141

[26] Nordon C, Karcher H, Groenwold RHH, Ankarfeldt MZ, Pichler F, Chevrou-Severac H, et al. The “Efficacy-Effectiveness Gap”: historical background and current conceptualization. Value Health. 2016;19:75–81. 26797239 10.1016/j.jval.2015.09.2938

[27] Wilson BE, Booth CM. Real-world data: bridging the gap between clinical trials and practice. eClinicalMedicine [Internet]. 2024 [cited 2025 Jun 25];78. Available from: https://www.thelancet.com/journals/eclinm/article/PIIS2589-5370(24)00494-2/fulltext. 10.1016/j.eclinm.2024.102915 PMC11585814 39588211

[28] Thompson D. Replication of randomized, controlled trials using real-world data: What could go wrong?. Value Health. 2021;24:112–5. 33431143 10.1016/j.jval.2020.09.015

[29] Carelli V, Carbonelli M, de Coo IF, Kawasaki A, Klopstock T, Lagrèze WA, et al. International consensus statement on the clinical and therapeutic management of Leber hereditary optic neuropathy. J Neuroophthalmol. 2017;37:371. 28991104 10.1097/WNO.0000000000000570

